# The Hard Choice about Dry Pet Food: Comparison of Protein and Lipid Nutritional Qualities and Digestibility of Three Different Chicken-Based Formulations

**DOI:** 10.3390/ani12121538

**Published:** 2022-06-14

**Authors:** Nicolò Montegiove, Eleonora Calzoni, Alessio Cesaretti, Roberto Maria Pellegrino, Carla Emiliani, Alessia Pellegrino, Leonardo Leonardi

**Affiliations:** 1 Department of Chemistry, Biology and Biotechnology, Biochemistry and Molecular Biology Section, University of Perugia, Via del Giochetto, 06123 Perugia, Italy; eleonoracalzoni@gmail.com (E.C.); alex.cesaretti14@gmail.com (A.C.); roberto.pellegrino@unipg.it (R.M.P.); carla.emiliani@unipg.it (C.E.); 2 Centro di Eccellenza sui Materiali Innovativi Nanostrutturati (CEMIN), University of Perugia, Via del Giochetto, 06123 Perugia, Italy; 3 Independent Researcher, Via Indipendenza 31/A, 06081 Assisi, Italy; alessiapellegrino@befood-pet.com; 4 Department of Veterinary Medicine, University of Perugia, Via San Costanzo 4, 06126 Perugia, Italy; leonardo.leonardi@unipg.it

**Keywords:** dry pet food, kibbles, soluble protein content, protein bioavailability, crude protein content, essential amino acids, crude fats, monounsaturated fatty acids, polyunsaturated fatty acids, digestibility

## Abstract

**Simple Summary:**

The majority of pet food currently on the market is represented by dry food thanks to its practicality and long shelf life. Dry pet food production consists of several processes that can have different effects on nutrient bioavailability and digestibility. The aim of this study was to analyze the nutritional quality of three different chicken-based formulations, consisting of fresh meats, meat meals, or a mix of these two from a protein, lipid, and in vitro digestibility point of view. The results show that the fresh chicken-meat-based formulation appears to be the preferable choice when proteins, lipids, and in vitro digestibility are taken into account. Moreover, the soluble protein content estimated by the Bradford assay is found to correlate well with the total protein content and in vitro digestibility.

**Abstract:**

Dry pet food, made of fresh meats and especially meat meals, represents one of the main types of complete food available on the market by virtue of its practicality and long shelf life. The kibble production process includes mixed thermal and mechanical treatments that help to improve the palatability and durability of the final product but may have undesirable effects on nutrient bioavailability and digestibility. An analysis of the protein and lipid content of different dry pet food formulations, together with an in vitro digestibility analysis, can reveal which formulation can provide a more nourishing diet for pets. In this study, a quantitative and qualitative analysis was performed on three different formulations of chicken-based dry pet food, consisting of fresh meats, meat meals, or a mix of these two. The soluble protein concentration was determined by the Bradford assay, while the crude protein content was assessed through the Kjeldahl method. Quadrupole time-of-flight liquid chromatography/mass spectrometry (Q-TOF LC/MS) was used to analyze the amino acid (AA) and lipid compositions. Finally, a gastric and small intestinal digestion simulation was used to determine the in vitro digestibility. The results show that dry pet food consisting only of chicken fresh meats has the highest content of soluble protein; it also contains more Essential AAs, Branched-Chain AAs, and Taurine, as well as a greater quantity of monounsaturated and polyunsaturated fatty acids. In addition, its in vitro digestibility was the highest, exceeding 90% of its dry weight, in agreement with the soluble protein content. These findings thus make the fresh-meat-based formulation a preferable choice as dry pet food.

## 1. Introduction

Pet food is generally grouped into wet, semi-wet, or dry based on the moisture content. The respective amount of water contained in the food is usually less than 11% in dry pet food, and its percentage varies from 25 to 35% in semi-wet foods, while in the wet ones, it can vary from 60 to 87% [1,2,3]. Dry pet food is often more appreciated by pet owners thanks to its practicality, low cost, ease of use, and long shelf life; additionally, it is possible to have food that is already formulated according to the nutritional requirements of pets, taking into account their age, size, and state of health [3,4,5,6,7,8]. Nowadays, complete foods that can be found on the market are formulated in such a way as to meet different animals’ needs (growth, maintenance, or gestation); in fact, by combining different proteins, fats, carbohydrates, vitamins, and minerals, it is possible to obtain a mix of formulations able to satisfy various nutritional needs [3]. The raw materials used as a source of proteins for the production of dry pet foods are principally represented by meat meals, but fresh meats are also widely used, and both of them can provide the desired amount of crude protein and the proper balance of amino acids (AAs) to fulfill the animal’s nutrient requirements [9,10,11,12,13]. Meat-based meals are made from the transformation of meat by-products in accordance with Regulation (EC) No. 1069/2009 of the European Parliament and of the Council of 21 October 2009, while fresh meats are derived from the slaughtering waste of meat intended for human use and are not subjected to particular technological processes, resulting in a smaller loss of nutrients [14]. Meat meals, on the other hand, are obtained by the rendering process, a cooking procedure that allows the separation and removal of the fatty portion, with the remaining part being subsequently dried [15,16]. According to Regulation (EC) No. 1069/2009, these nonmeat raw materials collected during slaughter processes may include animal horns, hooves, feathers, and bristles, which contain large amounts of fibrous proteins such as collagen, keratin, and elastin, which are insoluble or poorly soluble and often resistant to digestion compared to globular proteins, which tend to be more soluble and digestible [1,10,17,18]. Furthermore, the harsh industrial process to which meat meals are subjected may result in oxidation and partial deterioration of the raw components [19,20,21,22,23]; as a consequence, the quality of the final product obtained is often poor, as it strictly depends on the quality of the ingredients used [15,24,25,26].

The dry pet food production process consists of several steps, such as thermal and mechanical treatments, which, in addition to sanitizing food matrices, help to improve the nutritional properties, the bioavailability of nutrients, and the shelf life of the final product [2,22,27,28]. Dry pet foods are generally obtained by the extrusion process; this is a rapid cooking process that uses a combination of heat, pressure, and steam to sterilize the starting raw materials, obtain an expansion of the final shape of the product, and break the bonds of starch, allowing it to gelatinize, melt, and degrade, since starch is a primary ingredient widely used in dry pet food production [2,22,29]. More specifically, the raw materials are brought to a temperature between 100 °C and 200 °C in a few seconds, thanks to which the food is sterilized, enzymes are inactivated, and anti-nutritional factors are destroyed [3,20,22,30,31,32]. In addition, during the final step of production, fats are added to kibbles in a coating process in order to make them more palatable to pets. The most commonly used fats for the final coating of dry pet food are animal fats, i.e., chicken and pork fats [33].

As a result of the heat treatment, extrusion can affect the nutritional and organoleptic characteristics of the final product [2,22]. It can cause protein denaturation, alterations in the structures of carbohydrates, the oxidation of lipids, and the occurrence of the Maillard reaction responsible for the browning of the product and the formation of volatile substances, all processes that can alter the final characteristics of the product, such as the color, flavor, and aroma [22,30]. Industrial processes, therefore, affect the lipid component, subject to phenomena of hydrogenation, isomerization, and, in particular, oxidation, a process that can also occur during product storage and which is strictly dependent on the nature of the lipids and the degree of humidity [22,34].

The protein content is certainly a key ingredient of the final product for pets; in fact, the composition of AAs and the bioavailability of proteins define an important nutritional value of the final product [22,35], but it can be altered by production processes [22,29,32,36]. As far as the proteins are concerned, during the extrusion treatment, high temperatures can alter their structure; however, this event is not exclusively negative because slight protein denaturation can favor their subsequent digestion by the body and therefore improve their digestibility [22,36,37]. The undesirable effects of heat treatment also involve the destruction of AAs and the racemization process, which appears to have negative effects on the food’s nutritional characteristics since it is well known that only the levogyre form is the biologically active one [29,38].

Another important parameter that can be altered during dry pet food production is the level of Taurine, a sulfur-containing AA. This compound is of fundamental importance in pet diets, as it is involved in the transmission of nerve impulses, the synthesis of bile acids, and reductions in muscle damage and oxidative stress, supporting physical recovery after an intense effort [39,40]. Most animal tissues need a high concentration of Taurine, particularly the muscles, viscera, and brain, but not all pets are able to synthesize it. Therefore, the dietary intake of Taurine is essential for maintaining its normal concentration in the body [22]. An inadequate supply of Taurine can cause very serious pathological states, mainly for cats, which cannot synthesize it and thus may develop retinal degeneration and cardiomyopathies [41,42,43,44].

Hence, the aim of this work was to perform a quantitative and qualitative analysis of three different formulations of chicken-based complete dry pet food. In particular, dry pet foods prepared starting from only Chicken Fresh Meats (CFMs), a Mix of Chicken Fresh Meats and Chicken Meat Meals (CMix), and a formulation consisting of only Chicken Meat Meals (CMMs) were analyzed. Chicken-based dry pet food was chosen by virtue of its high-quality protein source, additionally characterized by high digestibility [16,18]. A soluble protein content analysis, which represents a convenient digestibility index [10,13,17], was carried out using the Bradford assay [45]. At the same time, the crude protein content was evaluated through the Kjeldahl method [46,47,48]. Moreover, a quantitative analysis of the different samples by quadrupole time-of-flight liquid chromatography/mass spectrometry (Q-TOF LC/MS) was carried out in order to evaluate the AA profile, with particular attention to the concentration of Essential AAs (EAAs) and Branched-Chain AAs (BCAAs).

The lipid content of the three different formulations was also assessed, starting with the evaluation of crude fats by the gravimetric method [46]. The lipid profile was then evaluated by Q-TOF LC/MS to determine the fatty acid (FA) content, expressly measuring the concentrations of monounsaturated (MUFAs), polyunsaturated (PUFAs), and saturated FAs (SFAs).

In the end, the digestibility of the different types of formulations used in this study was analyzed through an in vitro gastric digestion simulation followed by a small intestine digestion simulation [10].

The obtained results allow direct comparisons between the nutritional qualities of the three formulations analyzed, giving helpful guidelines about which formulation represents a preferable choice when it comes to dry pet food.

## 2. Materials and Methods

### 2.1. Raw Materials

The dry pet foods used in this study consist of: a formulation of kibble made only from Chicken Fresh Meats (CFMs) for companion animal food, 12 batches produced by an Italian pet food manufacturer; a formulation of kibble made from a Mix of Chicken Fresh Meats and Chicken Meat Meals 1:1 (*w*/*w*) (CMix) for companion animal food, 12 batches produced by an Italian pet food manufacturer; and a formulation of kibble made only from Chicken Meat Meals (CMMs) for companion animal food, 12 batches produced by an Italian pet food manufacturer. The chicken meal was obtained from necks, wings, and carcasses without legs. Each formulation also contains different percentages of the following ingredients based on the amount of chicken meat and/or chicken meal: dry rice, pre-cooked rice, sorghum, beetroot, hydrolyzed pork liver, hydrolyzed chicken proteins, and chicken fat. All the formulations were processed in the same way, and the same method of extrusion was employed.

### 2.2. Chemicals

Tris-HCl, IGEPAL^®^ CA-630, Bovine serum albumin (BSA), Sulfuric acid, Hydrochloric acid, Alanine, Arginine, Asparagine, Aspartic acid, Cysteine, Glutamic acid, Glycine, Histidine, Hydroxyproline, Isoleucine, Leucine, Lysine, Methionine, Phenylalanine, Proline, Serine, Taurine, Threonine, Tryptophan, Tyrosine, Valine, Diethyl ether, Petroleum ether, Butylated Hydroxytoluene, Methanol, Methyl tert-butyl ether, Chloroform, Sodium hydroxide, n-Hexane, Acetonitrile, Isopropyl Alcohol, Ammonium Acetate, Pepsin, Pancreatin, Trypsin, α-Chymotrypsin, Protease, Lipase, Disodium phosphate, Sodium bicarbonate, Sodium chloride, Potassium chloride, Magnesium chloride, Calcium chloride, and Bile salts were purchased from Sigma-Aldrich (Saint Louis, MO, USA).

Quick Start™ Bradford 1× Dye Reagent was purchased from Bio-Rad (Hercules, CA, USA).

### 2.3. Determination of Moisture Content

The moisture content was calculated according to the official method for the animal feed moisture analysis described by the AOAC [46]. Briefly, an exact amount of dry pet food (2 g) was shredded, evenly distributed on a dish, and finally dried in an oven (Termaks TS 8136, Bergen, Norway) at 135 °C for 2 h. Samples were cooled down at room temperature in a desiccator containing silica gel and weighed using an OHAUS Pioneer™ Analytical Balance (OHAUS Corporation, Parsippany, NJ, USA) until a constant and stable weight was reached. Water content was calculated as the difference between the initial and final weights.

### 2.4. Protein Solubilization

Pet kibbles were diluted in a hypotonic solution (10 mM Tris-HCl pH 7.5) at a concentration of 30 g/L (*w*/*v*) and homogenized using ULTRA-TURRAX T25 (IKA^®^-Werke GmbH & Co. KG, Staufen, Germany) for 90 s at 4 °C. After that, 0.1% (*v*/*v*) IGEPAL^®^ CA-630 was added to facilitate protein release from the organic matrix. Samples were then sonicated for 30 s at 4 °C using an ultrasonic disintegrator (Soniprep 150, MSE, Heathfield, East Sussex, UK). Finally, centrifugation at 10,000× *g* for 5 min at 4 °C was carried out to remove the insoluble material. The soluble fraction containing soluble proteins was recovered and used for the Bradford assay.

### 2.5. Determination of Soluble Proteins

The content of soluble proteins in the samples was assessed by the Bradford assay [45] using Quick Start™ Bradford 1× Dye Reagent (Bio-Rad, Hercules, CA, USA) according to the manufacturer’s instructions for one-step determination of protein concentration. The quantitative determination was carried out using the Coomassie Brilliant Blue G-250 dye (Bio-Rad, Hercules, CA, USA), which has an absorption peak at 595 nm in the protein-bound form. The absorbance was measured at 595 nm using a Shimadzu UV-160A UV-Visible Recording Spectrophotometer (Shimadzu Scientific Instruments, Kyoto, Japan). The concentration of the soluble proteins in the samples was obtained from their absorbance using a calibration curve prepared with known concentrations of bovine serum albumin (BSA; Sigma-Aldrich, Saint Louis, MO, USA). Each sample was analyzed in triplicate. Data are expressed as g of soluble protein per 100 g of dry sample.

### 2.6. Determination of Nitrogen Content

The Kjeldahl method was used to assess the sample nitrogen content according to the official method [46,47,48]. Briefly, the same amount of dry pet food (1 g) was digested in sulfuric acid in the presence of a catalyst at 420 °C for 1 h to convert the amine nitrogen to ammonium ions. The latter were then transformed into ammonia, which can be separated from the digestion mixture using a distiller UDK 129 (VELP Scientifica, Usmate, MB, Italy). Finally, the ammonia concentration was quantified by titration with a standard solution of hydrochloric acid. Once the total nitrogen content in the samples was determined, the crude protein content was calculated using a conversion factor of 6.25, since most meat proteins contain about 16% nitrogen [49]. Each sample was analyzed in duplicate. Data are expressed as g of crude protein per 100 g of dry sample.

### 2.7. Amino Acid Profile

The amino acid (AA) profiles of the three different kibble formulations were assessed using Q-TOF LC/MS. The hydrolysis of the dry pet food was carried out according to the method suggested by Otter et al. [50] with minor modifications. In brief, to obtain a solution of AAs from kibbles, 2 g of dry sample was suspended in 30 mL of 6 M HCl. Then, the solution was digested for 24 h at 110 °C using a water-cooled reflux condenser placed on a boiling flask. This temperature allows rapid hydrolysis of the protein component with mean recovery values of AAs greater than 98%; however, Cysteine cannot be detected under these conditions [50,51]. Furthermore, acid hydrolysis can lead to the conversion of Glutamine and Asparagine into Glutamic and Aspartic acid [51]. Moreover, the method of analysis used can inherently lead to an underestimation of Methionine, Serine, Threonine, and Tryptophan, which can be destroyed during the acid hydrolysis reaction [52]. The AAs considered in this study were: Alanine, Arginine, Asparagine, Aspartic acid, Cysteine, Glutamic acid, Glycine, Histidine, Hydroxyproline, Isoleucine, Leucine, Lysine, Methionine, Phenylalanine, Proline, Serine, Taurine, Threonine, Tryptophan, Tyrosine, and Valine.

After the hydrolysis step, an aliquot of 50 μL of the hydrolyzed mixture was diluted with distilled water and filtered through a C18 solid-phase extraction (SPE) cartridge for the defatting step. The ion-pairing chromatography (IPC) method was used to achieve a wide separation of AAs with a 150 × 2.1 mm, 3 μm ACME^TM^ Amide C18 column (Phase Analytical Technology LLC, State College, PA, USA) thermostated at 50 °C. The separation of AAs was achieved using a flow of 0.35 mL/min of a binary gradient of 0.3% heptafluorobutyric acid in water (solvent A) and 0.1% formic acid in methanol (solvent B). The initial condition was 2% B for 2 min, followed by a gradient from 2 to 80% B in 5 min, and a final isocratic step of 8 min.

The spectrometer was operated in high-resolution full-scan mode, monitoring positive ions. The quantitative data were obtained by external calibration in the range 0.05–2.5 μg/mL of a homemade mix of AAs in pure methanol. In the end, 1 μL of the sample was loaded into an Agilent 6530 Q-TOF LC/MS instrument (Agilent Technologies, Inc., Santa Clara, CA, USA) for AA profile analysis. Each sample was analyzed in duplicate. Data are expressed as g of AA per 100 g of dry sample.

### 2.8. Determination of Crude Fat Content

The determination of the total fat content was carried out by the gravimetric method [46]. In brief, an amount corresponding to 2 g of each dry sample was first hydrolyzed by hydrochloric acid and then extracted by a combination of diethyl ether and petroleum ether. Solvents were then placed into a pre-weighed conical flask, letting them decant, and evaporated by placing the flask on a steam bath. Finally, the samples were dried in an oven (Termaks TS 8136, Bergen, Norway) at 100 °C for 90 min. After cooling down at room temperature, the weight of the pre-weighed conical flask containing fats was recorded, and crude fat content was calculated. Each sample was analyzed in triplicate. Data are expressed as g of crude fat per 100 g of dry sample.

### 2.9. Lipid Profile

For the lipid extraction [53], a quantity corresponding to 100 mg of each dry sample was carefully weighed in a tube, and 1 mL of a 10 mM butylated hydroxytoluene solution in a mixture of methanol/methyl tert-butyl ether/chloroform (1:1:1) was added. The samples were then shaken for 30 min at 1500 rpm at room temperature in a Thermomixer T-Shaker (EuroClone, Pero, Italy). Subsequently, the samples were centrifuged at 1500× *g* for 10 min at room temperature using an Eppendorf 5418 centrifuge (Eppendorf, Hamburg, Germany). The supernatant containing the lipid fraction of the sample was then recovered. To release the fatty acid (FA) component of glycerolipids and phospholipids, strong basic hydrolysis was performed. An aliquot of 100 μL of the supernatant, obtained as described above, was transferred into a 2 mL Eppendorf Safe-Lock tube (Eppendorf, Hamburg, Germany) with 80 μL of a freshly prepared solution of 2% NaOH in methanol/water at a concentration ratio of 8:2 (*v*/*v*). The tube was shaken and heated in a Thermomixer T-Shaker (EuroClone, Pero, Italy) at 60 °C for 30 min. Afterward, the solution was cooled at room temperature and acidified with 20 μL of 12 M HCl, and 1 mL of n-hexane was added. The tube was vortexed for 10 s and centrifuged at 1500× *g* for 5 min. In the end, 250 μL of the supernatant, containing all FAs, was transferred to an autosampler vial for subsequent analysis. LC/MS analysis was conducted using an Agilent 6530 Q-TOF LC/MS instrument (Agilent Technologies, Inc., Santa Clara, CA, USA). FAs were separated using a Kinetex C18 column (4.6 mm × 100 mm, 2.6 µm, Phenomenex Inc., Aschaffenburg, Germany) with a 15 min linear gradient from 40% to 90% acetonitrile/water 60:40 (*v*/*v*) (solvent A) and isopropyl alcohol (solvent B), both containing 10 mM ammonium acetate. The column operated at 20 °C with a flow of 0.8 mL/min. Liquid chromatography was interfaced to a mass spectrometer with an Agilent JetStream source. The mass spectrometer acquired negative ions in full-scan mode in the mass range of 100–1700 with an accuracy of 1.5 ppm. This was achieved by continuous infusion in the source of a reference mass solution (G1969-85001, Agilent Technologies, Inc., Santa Clara, CA, USA). LC/MS raw files were aligned and processed using the Batch Recursive Feature Extraction algorithm of the Agilent MassHunter Profinder software (version B.08.00) (Agilent Technologies, Inc., Santa Clara, CA, USA). Afterward, data with a score > 90% were imported into the Agilent Mass Profiler software (version B.08.01) (Agilent Technologies, Inc., Santa Clara, CA, USA). The FA database was downloaded from LIPID MAPS^®^ Structure Database (LMSD) [54] and adapted to work in Agilent Mass Profiler software. Only FAs with a score > 90% were retained. At the end of the workflow, a data matrix reporting the abundance of the peaks of 40 FAs (9 saturated, 7 monounsaturated, and 24 polyunsaturated) was created and used to determine lipid content. Each sample was analyzed in duplicate. Data are expressed as g of FA per 100 g of dry sample.

### 2.10. In Vitro Digestibility

Digestibility was assessed according to a method previously described [10].

Gastric digestion simulation: Samples were finely ground (<1 mm particle size). A quantity corresponding to 500 mg of dry matter was weighed for each sample, taking into account the moisture content previously measured. Each sample was incubated with 20 mL of a pepsin–HCl solution (0.075 N HCl, pepsin from porcine gastric mucosa 2 g/L) in a 50 mL tube in a shaking water bath at 39 °C for 2 h.

Small intestine digestion simulation: First, the pH level was adjusted to 7.5 with 1 N NaOH. Then, 20 mL of 10 g/L pancreatin from porcine pancreas, 1.6 g/L trypsin from porcine pancreas, 3.1 g/L α-chymotrypsin from bovine pancreas, 1.3 g/L protease from *Streptomyces griseus*, and 1 g/L lipase from *Rhizopus oryzae* dissolved in phosphate-buffered solution pH 7.5 (3.92 g NaHCO_3_, 3.72 g Na_2_HPO_4_, 0.23 g KCl, 0.19 g NaCl, 0.12 g MgCl_2_, and 0.08 g CaCl_2_ in 1 L of distilled water; [55]) was added to each tube. Immediately prior to the addition of the enzymatic solution, bile salts (50% cholic acid sodium salt and 50% deoxycholic acid sodium salt) were added to each tube at a final concentration of 25 g/L. Finally, the tubes were placed in a shaking water bath at 39 °C for 4 h.

Collection of the undigested fraction: After enzymatic digestion, the preparation was centrifuged (3000× *g* for 10 min at 4 °C), washed twice with distilled water, and re-centrifuged (3000× *g* for 5 min at 4 °C). The undigested residue was dried at 135 °C until constant weight.

In order to determine the dry matter digestibility of the samples, the residue obtained from each tube after the in vitro digestion was weighed, and the digestibility was calculated with the following equation:

In vitro digestibility % = 100 − [(dry residue weight × 100)/dry sample weight]

### 2.11. Statistical Analysis

All data shown in this study regarding the analysis of the protein and lipid contents and the in vitro digestibility of the three different kibble formulations used for the production of dry pet food are reported as mean values of the twelve analyzed batches ± standard error of the mean (SEM). The one-way ANOVA test was employed to investigate the significance of the protein and lipid content differences between the means of each type of formulation. The test was also used to evaluate the significance of the differences in the AA content and in the in vitro digestibility between the three different formulations. The level of significance for the data was set at *p* < 0.05. Pearson’s correlation analysis was used to highlight the possible relation between the kibble protein content obtained with two different methods (Bradford assay vs. Kjeldahl method) and between the dry pet food in vitro digestibility and the protein content assessed by the Bradford assay and the Kjeldahl method. All statistical tests were performed using GraphPad Prism 9.00 for Windows (GraphPad Software, San Diego, CA, USA).

## 3. Results

### 3.1. Protein Content

The moisture level of each formulation was assessed before evaluating the soluble protein content by the Bradford assay. The results shown in Table 1 reveal that CFM and CMM formulations exhibit higher water content compared to the CMix formulation. The humidity level in CFM and CMM formulations is about 8%, whereas a water content lower than 6% is unique to the CMix formulation.

The Bradford assay was then performed on the three different kibble formulations for pets, taking into account the different water content, and revealed that the CFM formulation contains a higher level of soluble proteins as compared to CMix and CMM formulations (Table 1). The meat-based formulation has a quantity of soluble proteins of 8.9 g per 100 g of dry sample, while the other formulations have an amount more than halved compared to the CFM formulation, 4.0 g per 100 g of dry sample for CMix and 2.4 g per 100 g of dry sample for the CMM formulation. The statistical analysis conducted on all of the different kibble formulations revealed statistically significant differences (*p* < 0.0001) between the soluble protein content of CFM, CMix, and CMM formulations.

The crude protein content was also evaluated through the Kjeldahl method. The results shown in Table 1 reveal that the CFM formulation has the highest quantity of crude protein, 26.40 g per 100 g of dry sample, followed by the mix (23.77 g/100 g of dry sample) and meal-based (22.7 g/100 g of dry sample) formulations. The statistical analysis performed on all of the different kibble formulations revealed statistically significant differences (*p* < 0.0001) between the crude protein content of CFM, CMix, and CMM formulations.

**Figure 1:**
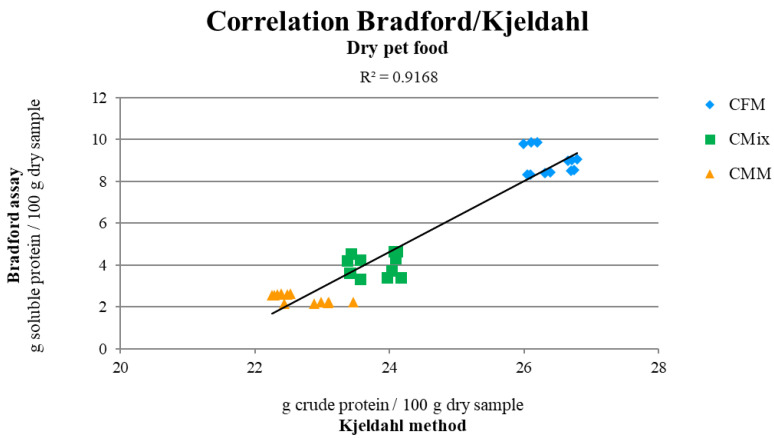
Correlation between the soluble and crude protein contents of CFM, CMix, and CMM formulations determined by the Bradford assay and the Kjeldahl method. Data are reported as mean, n = 12.

The correlation between the two methods for estimating protein content was also investigated, and when the data are compared, it emerges that the Bradford assay and the Kjeldahl method have a direct correlation (R^2^ = 0.9168) (Figure 1), which is highly dependent on the composition of the dry pet food formulation. In fact, average factors of 2.97, 6.05, and 9.51 are found between the values of the Bradford assay and the Kjeldahl method obtained for CFM, CMix, and CMM formulations, respectively, corresponding to the ratio between the average values of the protein content found with the two methods.

### 3.2. Amino Acid Profile

The amino acid (AA) composition of the three different kibble formulations for companion animal food was analyzed through acid hydrolysis, followed by Q-TOF LC/MS analysis. The results indicate that the amount of total AAs in the CFM formulation is higher than in the other formulations, with 20.6 g of AAs per 100 of a dry sample, while in CMix and CMM formulations there are respectively 18.2 g and 17.9 g of AAs per 100 of a dry sample (Table 2).

The statistical analysis performed on all of the different kibble formulations showed statistically significant differences (*p* < 0.0001) between the total AA content in CFM, CMix, and CMM formulations.

All of the AAs detected are shown in Table 2. The analysis showed that the CFM formulation is significantly enriched in all of the EAAs for pets (10.9 g of EAAs/100 of a dry sample). This formulation shows a higher quantity of Arginine, Histidine, Isoleucine, Leucine, Lysine, Methionine, Phenylalanine, Threonine, Tryptophan, and Valine than CMix (9.4 g of EAAs/100 of a dry sample) and CMM (8.9 g of EAAs/100 of a dry sample) formulations. Additionally, the BCAA content is higher in the CFM formulation (4.4 g of BCAAs/100 g dry sample) than in CMix (4.0 g of BCAAs/100 g dry sample) and CMM (3.9 g of BCAAs/100 g dry sample) formulations (Table 2).

In addition, CFM kibbles are found to be richer in Taurine, with 201 mg of Taurine/100 g dry sample, compared to the other formulations: 162 mg per 100 g of dry sample for the CMix formulation and 134 mg per 100 g of dry sample for the CMM formulation.

The statistical analysis (Table 2) showed that the observed differences between the AA content of CFM, CMix, and CMM formulations are, for the most part, statistically significant. In particular, most of the EAAs for pets are significantly more abundant in the formulation obtained from chicken meat alone.

### 3.3. Determination of Crude Fat Content

**Figure 2:**
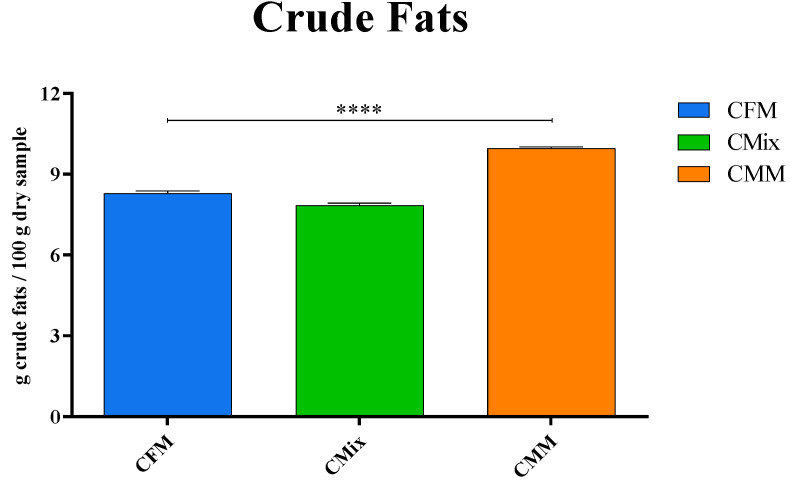
The crude fat content of CFM, CMix, and CMM formulations for companion animal food. Data are reported as mean ± SEM, n = 12. **** *p* < 0.0001.

The crude fat content of each formulation was evaluated according to the official method described in the Materials and Methods section. The results shown in Figure 2 represent the average of the crude fat values obtained for each dry pet food formulation analyzed. The analysis revealed that the CMM formulation exhibits a significantly higher crude fat content compared to the other formulations. The statistical analysis of the different crude fat contents in the three different formulations showed statistically significant differences (*p* < 0.0001) between the three formulations.

The crude fat level, reported as weight percentage with respect to the dry sample, ranges from 9.97 g of crude fats per 100 g of dry sample in the case of the CMM formulation to 8.29 g of crude fats per 100 g of dry sample in the case of the CFM, with 7.84 g of crude fats per 100 g of dry sample in the case of the CMix formulation.

**Figure 3:**
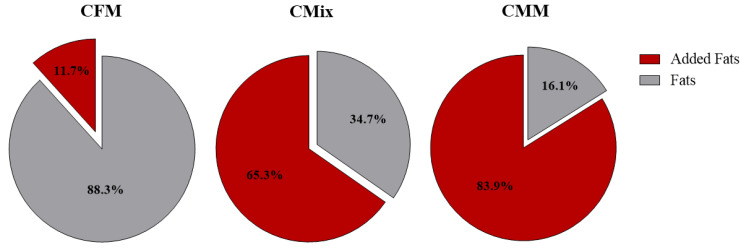
Crude fat composition, expressed as percentages, of CFM, CMix, and CMM formulations for companion animal food. Data are reported as mean, n = 12.

However, the percentage of chicken fats added in the final step of kibble production turns out to be dissimilar in the different formulations. In particular, it was found that 0.97 g of fats per 100 g of dry sample was added in the CFM kibbles, while 5.12 g in the CMix formulation and 8.36 g in the CMM formulation. The lipid component of the different formulations was therefore enriched in fats during the final production phase, with 11.7% with respect to the total lipid content as far as the CFM formulation is concerned, 65.3% in the CMix formulation, and 83.9% in the CMM formulation (Figure 3).

### 3.4. Lipid Profile

**Figure 4:**
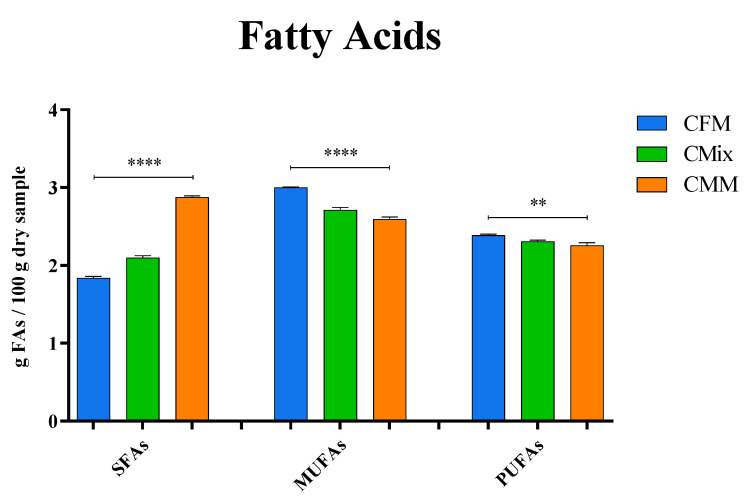
FA content of CFM, CMix, and CMM formulations for companion animal food. Data are reported as mean ± SEM, n = 12. ** *p* < 0.01, **** *p* < 0.0001.

The fatty acid (FA) content in each formulation was evaluated through Q-TOF LC/MS. The data reported in Figure 4 show the quantity of saturated (SFAs), monounsaturated (MUFAs), and polyunsaturated FAs (PUFAs) present in the formulations analyzed.

The results obtained are expressed as g of FAs per 100 g of dry sample. The content of SFAs is significantly higher in the CMM formulation, while the concentrations of MUFAs and PUFAs are higher in the CFM formulation compared to the others. The concentration of SFAs in the CMM formulation is found to be about 2.88 g of SFAs per 100 g of dry sample, followed by CMix (2.10 g of SFAs/100 g of dry sample) and CFM formulations (1.84 g of SFAs/100 g of dry sample), which shows the lowest concentration. As far as MUFAs are concerned, the highest concentrations are instead found for the CFM formulation (3.00 g of MUFAs/100 g of dry sample), followed by CMix (2.71 g of MUFAs/100 g of dry sample) and CMM (2.60 g of MUFAs/100 g of dry sample) formulations. As regards PUFAs, their content is also found to be the highest in the CFM formulation, 2.39 g of PUFAs per 100 g of dry sample, followed by CMix (2.31 g of PUFAs/100 g of dry sample) and CMM (2.26 g of PUFAs/100 g of dry sample) formulations. The statistical analysis performed on all of the different kinds of kibbles showed statistically significant differences (*p* < 0.01, *p* < 0.0001) between the SFA, MUFA, and PUFA contents of CFM, CMix, and CMM formulations.

**Figure 5:**
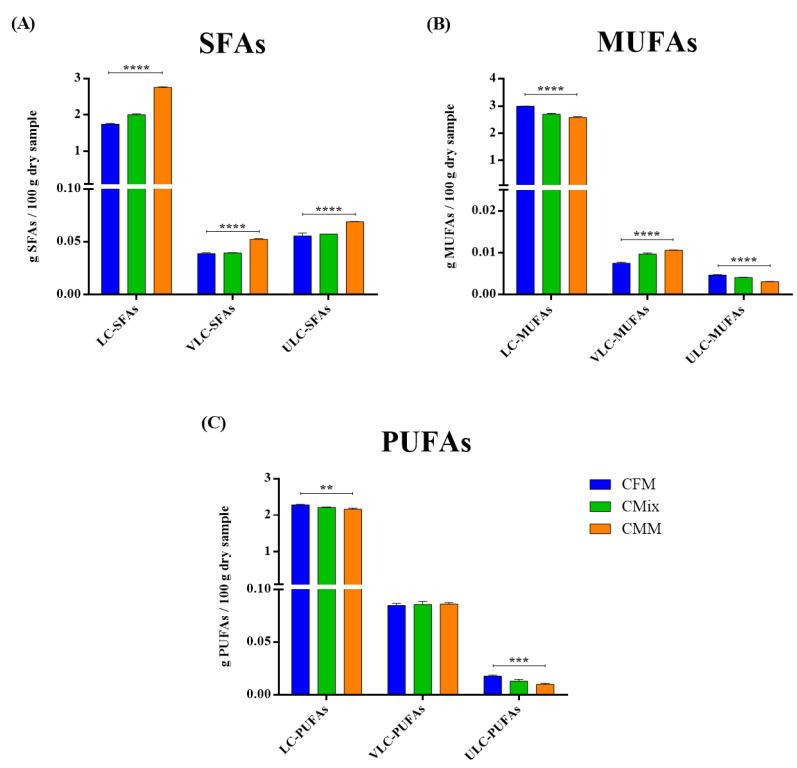
(**A**) LC-SFA, VLC-SFA, and ULC-SFA contents of CFM, CMix, and CMM formulations for companion animal food. (**B**) LC-MUFA, VLC-MUFA, and ULC-MUFA contents of CFM, CMix, and CMM formulations for companion animal food. (**C**) LC-PUFA, VLC-PUFA, and ULC-PUFA contents of CFM, CMix, and CMM formulations for companion animal food. Data are reported as mean ± SEM, n = 12. ** *p* < 0.01, *** *p* < 0.001, **** *p* < 0.0001.

Figure 5 reports the analysis of SFAs, MUFAs, and PUFAs, distinguishing them into Long-Chain (LC- 11 < C ≤ 20), Very Long-Chain (VLC- 20 < C ≤ 25), and Ultra-Long-Chain (ULC- C ≥ 26) FAs based on the length of the carbon chain [11].

As far as SFAs are concerned, the contents of LC-, VLC-, and ULC-SFAs are higher in the CMM formulation than in CFM and CMix kibbles, with 2.76 g of LC-SFAs, 52 mg of VLC-SFAs, and 69 mg of ULC-SFAs per 100 g of dry sample; the statistical analysis showed statistically significant differences (*p* < 0.0001) in LC-, VLC-, and ULC-SFA contents between the three formulations (Figure 5A). In the case of MUFAs, the contents of LC- and ULC-MUFAs are the highest in the CFM formulation (2.99 g of LC-MUFAs and 5 mg of ULC-MUFAs per 100 g of dry sample), whereas VLC-MUFAs are more abundant in the CMM formulation (11 mg of VLC-MUFAs per 100 g of dry sample). The statistical analysis highlighted statistically significant differences (*p* < 0.0001) between LC-, VLC-, and ULC-MUFA contents in CFM, CMix, and CMM formulations (Figure 5B). The contents of LC- and ULC-PUFAs are the highest in the CFM formulation (2.28 g of LC-PUFAs and 18 mg of ULC-PUFAs per 100 g of dry sample), while the content of VLC-PUFAs is comparable in all formulations (about 85 mg of VLC-PUFAs per 100 g of dry sample). The statistical analysis showed statistically significant differences (*p* < 0.01, *p* < 0.001) in LC- and ULC-PUFA contents but not in VLC-PUFAs (Figure 5C).

### 3.5. In Vitro Digestibility

**Figure 6:**
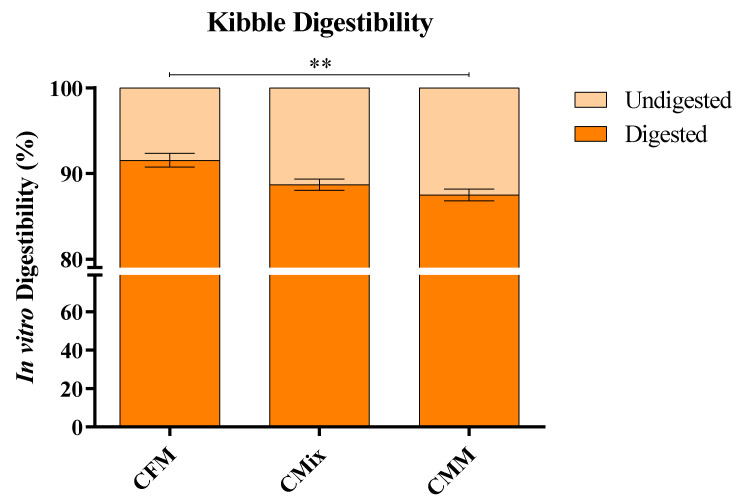
Digestibility of dry samples of CFM, CMix, and CMM formulations for companion animal food analyzed by the in vitro digestibility assay. Data are reported as mean ± SEM, n = 12. ** *p* < 0.01.

The digestibility of all dry pet food formulations was analyzed by in vitro gastric and small intestine digestion simulation. This analysis was carried out on the same amount of dry sample, and the indigested insoluble material was weighed at the end of the reaction. The results show that all of the formulations have a good degree of digestibility; however, the CFM formulation was significantly more digested following the in vitro digestion process compared to the other formulations (Figure 6). The amount of digested material is about 92% for the CFM formulation, about 89% for CMix, and only 87% for CMM. The statistical analysis performed on CFM, CMix, and CMM formulations highlighted statistically significant differences (*p* < 0.01) between the in vitro digestibility values of the different dry pet food formulations.

**Figure 7:**
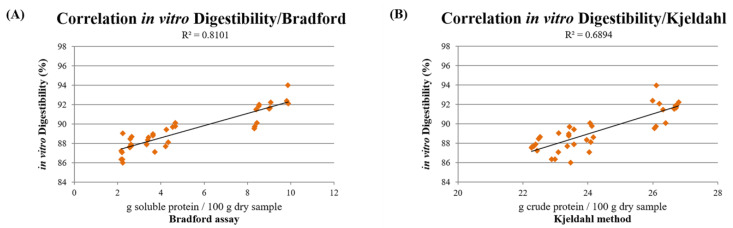
(**A**) Correlation between the in vitro digestibility assay and the soluble protein content evaluated by the Bradford assay in CFM, CMix, and CMM formulations for companion animal food. (**B**) Correlation between the in vitro digestibility assay and the crude protein content evaluated by the Kjeldahl method in CFM, CMix, and CMM formulations for companion animal food. Data are reported as mean ± SEM, n = 12.

The values of in vitro digestibility were then correlated with the results of the Bradford assay, revealing a linear correlation between the different dry pet food formulations’ soluble protein content and their in vitro digestibility (R^2^ = 0.8101) (Figure 7A). As far as the Kjeldahl method is concerned, the comparison with the digestibility values showed that there may be a correlation even in this case between the different kibbles’ crude protein contents and their in vitro digestibility, albeit weaker (R^2^ = 0.6894) (Figure 7B).

## 4. Discussion

Depending on the raw materials and the different industrial treatments used during the production phases, the final product can have different nutritional qualities [2,3,10,11,12,13,22,30]. Therefore, analyzing the nutritional qualities of dry pet food is essential to understanding which raw materials are preferable to use in order to obtain high-quality formulations that can guarantee the correct nutritional needs and good state of health of the animal.

The soluble protein contents of the three different formulations under investigation, initially assessed through the Bradford assay performed on the extracts of the final products after moisture evaluation (Table 1), show that the CFM formulation has a higher level of soluble proteins as compared to the other formulations (Table 1), making it a good candidate as the most digestible formulation, since previous studies have shown that a higher soluble protein content could be correlated with greater digestibility [10,17].

The different protein solubility in the different samples could be justified by the fact that the CMM raw materials include other edible animal parts besides skeletal meat containing fibrous proteins (e.g., collagen, elastin, and keratin), which exhibit less solubility and digestibility compared to globular proteins [17].

The analysis of the nitrogen contents of the three different kibble formulations carried out through the Kjeldahl method highlight that the CFM formulation has a significantly higher quantity of crude proteins (Table 1). These findings then show that the differences between the formulations are mainly in the soluble protein content; in fact, despite having similar nitrogen contents (above 20% of the dry weight of the sample), CFM exhibits the greatest quantity of soluble proteins, which are ultimately more bioavailable and digestible [10,17], suggesting that this could be the most desirable formulation among the three to use as pet food.

The presence of a correlation between the Bradford assay and the Kjeldahl method (R^2^ = 0.9168) (Figure 1), as regards the estimation of the protein content of final dry products, is in line with what has been observed in previous studies carried out on raw materials, which suggested the possible use of the Bradford assay for the estimation of the protein content in the dry pet food industry [10]. The correlation obtained is strictly dependent on the raw materials used in the dry pet food formulation process; in fact, different conversion factors were found depending on the raw materials considered (2.97 for CFMs, 6.05 CMix, and 9.51 for CMMs), so if this test is ever validated for its use in the dry pet food industry, it would need ad hoc conversion factors to bring the soluble protein content back to the total raw protein content value.

The total AA content of the different dry pet food formulations, assessed through acid hydrolysis followed by mass spectrometry analysis, as shown in Table 2, reveals that the CFM formulation exhibits a significantly higher AA content compared to the other formulations. The values of the total AAs of each formulation come close to the values found with the Kjeldahl method, suggesting that the nitrogen found in the previous analysis is almost entirely delegated to proteins and implying that, at the same time, there is a good recovery of the AA component following the acid hydrolysis process. However, the AA profile between the different formulations is different. In fact, a previous study conducted on raw materials showed that meat meals have a higher total AA content than fresh meat [10]. As far as the investigated final products are concerned, the opposite trend is observed (Table 2); this behavior could be explained by taking into account the different effects that the handling and storage processes have on the various raw materials. The CFM formulation shows the highest concentration for most EAAs, including BCAAs (Table 2). This is very important since the correct supply of AAs, particularly EAAs, is necessary to guarantee the good state of health of the animal, in that the lack of one or more of these AAs could cause problems, such as limiting effects on animal growth, leading to the onset of catabolic processes and the development of some diseases [56,57,58,59].

The concentration of Arginine is significantly higher in the CFM formulation (Table 2). This EAA is important, as it is involved, together with Glycine and Methionine, in the synthesis of Creatine, which, once phosphorylated, becomes a high-energy derivative that can transfer a phosphate group to ADP, resulting in the formation of the energy-giving molecule in living cells, i.e., ATP, that allows cellular reactions to be carried out. This EAA also has other functions, such as an antihypertensive effect in mammals; it helps decrease systolic pressure, prevents platelet aggregation, has tumoricidal and bactericidal effects and neurotransmitter functions, promotes wound healing, and induces the release of dopamine, and its systemic administration increases the levels of some plasma molecules, such as insulin, glucagon, and prolactin [58].

As is the case with Arginine, the right amount of Histidine is also fundamental in pet food. This EAA is again found to be significantly higher in the meat-based formulation (Table 2). Histidine is extremely important, especially for feline nutrition, as its deficiency contributes to stunted growth, anorexia, and cataract development in puppies [60]; furthermore, it can act as a free radical scavenger [58].

Isoleucine, Leucine, and Valine are also found to be significantly higher in the CFM formulation compared to the others (Table 2). These are EAAs with branched aliphatic side chains and are therefore defined as BCAAs [61,62]. Pets cannot synthesize these AAs, which must therefore be included in the diet, as they are involved in different metabolic pathways; play a key role in muscular endurance, muscle glucose uptake, insulin resistance, protein synthesis, and muscle growth; constitute about 35% of the muscle AAs; and are the ones initially degraded by muscles during their activity. They also contribute to energy homeostasis and the strengthening of the immune system [58,61,62,63,64,65]. Among the functions mentioned above, BCAAs also present a similar catabolic pathway, undergoing transamination to enrich the nitrogen pool in the body with the formation of Succinyl-CoA from Valine and Succinyl-CoA or Acetyl-CoA from Leucine and Isoleucine, important intermediates in the citric acid cycle [58].

As far as Lysine is concerned, the CFM formulation exhibits the highest concentration of this EAA as well (Table 2). Lysine is also considered a Limiting AA (LAA) and can negatively affect protein synthesis if its contribution is not sufficient [66,67]. Its requirement in puppies has been shown to increase as the dietary intake of total protein increases [67,68]. This EAA is also susceptible to heat treatments used for obtaining the final product; in fact, heat can induce a reaction between Lysine and reducing sugars, giving rise to the Maillard reaction, whose products are less digestible, can make food less palatable, and, above all, involve a reduction in the bioavailability of this EAA [67].

CFM kibbles also present the highest level of Methionine (Table 2), an EAA that, along with Lysine, is an LAA in most commercial pet foods [67]. In addition, it is involved in the synthesis of Creatine and plays a fundamental role in cat feeding, as it is a precursor of Taurine [58,69]. This sulfur-containing AA is also used to produce another sulfur-containing AA, i.e., Cysteine [67,70,71]. It has been shown that the supply of Methionine and Cysteine must be higher in cats than in dogs, as these AAs are used for the production of Felinine, a precursor of a particular pheromone [67,72].

Another EAA whose concentration is found to be significantly higher in the CFM formulation is Phenylalanine (Table 2). The latter is fundamental since L-Tyrosine, an important precursor of some neurotransmitters, is formed with the intervention of the Phenylalanine Hydroxylase enzyme, which uses Phenylalanine as a substrate [58,73]. The intake of this EAA with food is in fact very important as, in addition to being a fundamental constituent of proteins, it is also a precursor of Catecholamine neurotransmitters (e.g., L-DOPA, dopamine, noradrenaline, and adrenaline) [73]. It has also been shown that another pathway of L-Tyrosine and, therefore, Phenylalanine metabolism leads to the production of Melanin, a fundamental pigment for maintaining the color of the hair. In fact, appropriate intake of this EAA contributes, both in dogs and in cats, to the development and maintenance of optimal hair color [73,74,75].

As far as Threonine is concerned, although the CFM formulation has the highest mean quantity, there are no significant differences between the three kinds of dry pet food. All of the formulations have a comparable level of this EAA (Table 2), which is important, as it is a component of structural proteins, and from its metabolism is formed Pyruvate, which then enters the Krebs cycle [58]. Failure to provide this EAA causes weight loss and brain damage in pets [76,77].

The Tryptophan content was also analyzed, and its concentration is significantly higher in the CFM formulation than in CMix and CMM formulations (Table 2). This EAA, also considered an LAA, is fundamental for the health of pets as a precursor of Serotonin and of the vitamin Niacin; therefore, its intake is essential for the proper functioning of the nervous system [58,67].

Last but not least, Taurine was also found to have a significantly higher concentration in the CFM formulation (Table 2). This sulfur-containing AA is an extremely important nutrient for cats, as it represents an EAA, inasmuch they are not able to synthesize it, and its deficiency can cause serious health problems for the animal [41,42,43,44].

These results, therefore, highlight that the CFM formulation represents a preferable choice as dry pet food to ensure the correct supply of all of the EAAs to pets. This allows animals to synthesize the functional proteins required for normal physiological functions and to have more energy available, stronger musculature, and a healthier coat. The correct supply of EAAs with food also allows the synthesis of the metabolic intermediates essential for the functioning of the cell cycle.

As for the analysis of the lipid content, the CMM formulation is found to have a higher concentration of crude fats compared to CFM and CMix formulations (Figure 2). However, it is known that one of the final processes for the production of dry pet food involves the coating of fats, mainly of animal origin, i.e., chicken and pork fats, in order to make the kibble more palatable [33]. The analyses carried out to evaluate the lipid content before and after this phase of coating (chicken fats) have in fact shown that the percentage of fats added in the three different formulations is clearly different. In the case of the CMM formulation, about 83.9% of fats are added following the coating process, with about 65.3% in the CMix formulation and only 11.7% in the case of the CFM formulation (Figure 3). Since the raw materials consisting of fresh meat do not undergo the rendering process, to which meat meals are usually subjected and which also includes a degreasing phase, they are richer in fats, which therefore do not need to be added in excessive quantities during the final phases of production in order to make the kibbles more palatable.

The analysis of the lipid profile carried out by liquid chromatography coupled with mass spectrometry demonstrates that the CMM-based dry pet food formulation has a significantly higher content of SFAs, whereas the concentrations of MUFAs and PUFAs are significantly higher in the CFM formulation (Figure 4). While SFAs can show adverse effects on pet health, inducing insulin resistance, hepatocyte apoptosis, and lipotoxicity, resulting in inflammation [78], MUFAs and PUFAs show many beneficial effects on the health of pets, as they have anti-inflammatory properties, serve a structural role in biological membrane composition, improve the skin and coat, operate as prostaglandin and eicosanoid precursors, and can ultimately provide energy [11,79]. SFAs also increase plasma cholesterol concentrations in a dose-dependent manner, while MUFAs and PUFAs have the opposite effect [80]. Hence, the low SFA content and high MUFA and PUFA contents in CFM kibbles are positive points for this type of dry pet food preparation. These results also highlight that the crude fats constituting the CMM formulation are mostly composed of SFAs added during the fat coating process, which are ultimately more harmful to the animal’s health [78].

The analysis of the length of the lipid carbon chain also shows that in the CMM formulation, there is the highest content of LC-SFAs (Figure 5A), which could, however, have positive effects by limiting the serum concentration of cholesterol, but also higher contents of VLC and ULC-SFAs, which instead have the opposite effect [11,81,82]. On the other hand, in the case of MUFAs and PUFAs, LC-FAs are mainly present in the CFM formulation, which shows the highest concentrations (Figure 5B,C). This has positive effects on the health of pets, as there are studies that have shown that LC-MUFAs improve cardiovascular health, lower serum concentrations of cholesterol, and modulate immune functions [11,81,83,84]. LC-PUFAs also provide several benefits to animal health, having cardioprotective, immunoprotective, and anti-inflammatory effects [11,85].

As far as digestibility is concerned, the in vitro analysis revealed that all three formulations analyzed are digestible at about 90%. In particular, the most digestible formulation is CFM, with a percentage of digestibility greater than 90%, while the other two formulations are slightly less digestible (Figure 6). These results match those previously shown, inasmuch as CFM-based dry pet food has a greater quantity of soluble and therefore more digestible and bioavailable proteins than CMix and especially CMM formulations. In fact, the CMM formulation may have a lower digestibility than those containing fresh meats, probably due to their raw materials containing greater quantities of insoluble proteins, such as collagen or elastin, which are part of the connective tissue and are less digestible [10,17], as also evidenced by an in vivo study on rendered poultry by-product meals used in the pet food industry [16].

The presence of some degree of correlation (R^2^ = 0.8101) between the soluble protein content estimated by the Bradford assay and in vitro digestibility (Figure 7A) is in agreement with previous studies carried out on pet food raw materials and suggests that a method that discriminates the soluble protein component can also provide an estimate of the digestibility, although evaluated in vitro. Conversely, the correlation found between the Kjeldahl method and in vitro digestibility is weaker (R^2^ = 0.6894) (Figure 7B), ruling out the possibility of correlating the crude protein content with the in vitro digestibility of the different dry pet food formulations. However, it has to be noted that the involvement of other factors, such as the presence of fibers or anti-nutritional factors, as well as the processing and storage conditions, contributes to the different digestibility of the various final products investigated.

## 5. Conclusions

In this work, three different chicken-based dry pet food formulations were investigated from the protein, lipid, and in vitro digestibility point of view. The results obtained show that the dry pet food formulation consisting exclusively of CFMs has higher soluble and crude protein contents than the other two formulations analyzed, composed of CMix and CMMs. Moreover, a correlation between the two methods for estimating the protein content, i.e., the Bradford assay and the Kjeldahl method, emerged, confirming previous studies carried out on pet food raw materials. This study also shows that the CFM formulation has the highest content of EAAs, BCAAs, and Taurine, all important nutrients for pet health, suggesting that these kibbles are the desirable choice from a protein and AA content point of view.

As regards the lipid content, this work highlights that the CMM formulation has a higher crude fat content, mostly composed of SFAs added during the fat coating step, compared to CFM and CMix formulations, while the MUFA and PUFA contents are higher in the CFM formulation. These findings suggest that the latter is preferable from a lipid point of view, inasmuch as it has been amply demonstrated that these FAs play a crucial role in maintaining the optimal health of pets.

Lastly, the in vitro digestibility of the three different formulations shows that, in this case as well, the CFM formulation is preferred by virtue of its higher digestibility, probably due to its different soluble protein content, as the correlation with the Bradford method suggests.

In light of all of these results, the soluble protein content evaluated by the Bradford assay can be considered a factor that, albeit not commonly taken into account, could become a key factor in the food industry for the estimation of the total protein content, bioavailability, and digestibility, although only evaluated here in vitro, thanks to their correlations. Moreover, it is possible to conclude that CFM-based dry pet food represents the most suitable choice to satisfy the different needs of pets, from both the protein and lipid point of view, thus guaranteeing the most adequate and healthy nutritional protein and lipid intake for companion animals.

Further in vivo studies will, however, be needed to strengthen and confirm these preliminary results and to gain a greater understanding of the nutritional characteristics of different dry pet food formulations.

## Figures and Tables

**Table 1:** Moisture, soluble protein (SP) content determined by the Bradford assay, and crude protein (CP) content determined by the Kjeldahl method in CFM, CMix, and CMM formulations. Data are reported as mean ± SEM, n = 12. **** *p* < 0.0001.

Dry Pet Food	Type of Sample			
	CFM	CMix	CMM	*p*-Value
**Moisture** **(%)**	8.42 ± 0.05	5.43 ± 0.08	8.6 ± 0.1	****
**Bradford Assay** **(g SP/100 g Dry Sample)**	8.9 ± 0.2	4.0 ± 0.1	2.4 ± 0.4	****
**Kjeldahl Method** **(g CP/100 g Dry Sample)**	26.40 ± 0.09	23.77 ± 0.09	22.7 ± 0.1	****

**Table 2:** AA content in CFM, CMix, and CMM formulation for companion animal food evaluated by Q-TOF LC/MS analysis. Data are reported as mean, n = 12.

AA Content (g AA/100 g Dry Sample)	Type of Sample			
	CFM	CMix	CMM	*p*-Value
**Arginine**	1.48 ± 0.02	1.15 ± 0.01	1.02 ± 0.01	****
**Histidine**	0.536 ± 0.006	0.456 ± 0.005	0.425 ± 0.006	****
**Isoleucine**	1.19 ± 0.02	1.09 ± 0.02	1.07 ± 0.02	***
**Leucine**	2.22 ± 0.06	1.99 ± 0.01	1.94 ± 0.02	****
**Lysine**	2.40 ± 0.02	1.98 ± 0.01	1.82 ± 0.01	****
**Methionine**	0.320 ± 0.006	0.281 ± 0.003	0.212 ± 0.003	****
**Phenylalanine**	0.812 ± 0.009	0.751 ± 0.004	0.74 ± 0.01	****
**Taurine**	0.201 ± 0.009	0.162 ± 0.005	0.134 ± 0.004	****
**Threonine**	0.314 ± 0.005	0.298 ± 0.006	0.296 ± 0.007	ns
**Tryptophan**	0.49 ± 0.01	0.32 ± 0.01	0.286 ± 0.008	****
**Valine**	0.943 ± 0.005	0.935 ± 0.003	0.927 ± 0.003	*
**TOT EEAs**	**10.9 ± 0.2**	**9.4 ± 0.1**	**8.9 ± 0.1**	****
**Alanine**	1.53 ± 0.07	1.66 ± 0.07	1.99 ± 0.09	***
**Asparagine**	0.0221 ± 0.0004	0.0163 ± 0.0005	0.0140 ± 0.0003	****
**Aspartic acid**	2.26 ± 0.06	1.80 ± 0.01	1.95 ± 0.01	****
**Glutamic acid**	2.31 ± 0.06	1.78 ± 0.01	1.70 ± 0.03	****
**Glycine**	0.72 ± 0.05	0.67 ± 0.03	0.60 ± 0.02	ns
**Hydroxyproline**	0.571 ± 0.005	0.399 ± 0.003	0.308 ± 0.003	****
**Proline**	0.66 ± 0.01	0.614 ± 0.009	0.538 ± 0.008	****
**Serine**	0.74 ± 0.07	1.03 ± 0.08	1.07 ± 0.06	**
**Tyrosine**	0.91 ± 0.04	0.87 ± 0.01	0.84 ± 0.01	ns
**TOT non EAAs**	**9.7 ± 0.4**	**8.8 ± 0.2**	**9.0 ± 0.2**	**ns**
**TOT AAs**	**20.6 ± 0.5**	**18.2 ± 0.3**	**17.9 ± 0.3**	****

ns = difference is not statistically significant, * *p* < 0.05, ** *p* < 0.01, *** *p* < 0.001, **** *p* < 0.0001.

## Data Availability

Data is contained within the article.

## References

[1] Aldrich G. (2006). Rendered Products in Pet Food. Essent. Render..

[2] Gibson M.W., Sajid A. (2013). Pet Food Processing: Understanding Transformations in Starch during Extrusion and Baking. Cereal Foods World.

[3] Zicker S.C. (2008). Evaluating Pet Foods: How Confident Are You When You Recommend a Commercial Pet Food?. Top. Companion Anim. Med..

[4] Remillard R.L. (2008). Homemade Diets: Attributes, Pitfalls, and a Call for Action. Top. Companion Anim. Med..

[5] Morelli G., Stefanutti D., Ricci R. (2021). A Survey among Dog and Cat Owners on Pet Food Storage and Preservation in the Households. Animals.

[6] Di Donfrancesco B., Koppel K., Swaney-Stueve M., Chambers E. (2014). Consumer Acceptance of Dry Dog Food Variations. Animals.

[7] Rombach M., Dean D.L. (2021). It Keeps the Good Boy Healthy from Nose to Tail: Understanding Pet Food Attribute Preferences of US Consumers. Animals.

[8] Vinassa M., Vergnano D., Valle E., Giribaldi M., Nery J., Prola L., Bergero D., Schiavone A. (2020). Profiling Italian Cat and Dog Owners’ Perceptions of Pet Food Quality Traits. BMC Vet. Res..

[9] Thompson A. (2008). Ingredients: Where Pet Food Starts. Top. Companion Anim. Med..

[10] Montegiove N., Pellegrino R.M., Emiliani C., Pellegrino A., Leonardi L. (2021). An Alternative Approach to Evaluate the Quality of Protein-Based Raw Materials for Dry Pet Food. Animals.

[11] Montegiove N., Calzoni E., Cesaretti A., Alabed H., Pellegrino R.M., Emiliani C., Pellegrino A., Leonardi L. (2020). Comprehensive Evaluation of Lipidic Content in Dry Pet Food Raw Materials: Comparison between Fresh Meats and Meat Meals. Sci. Bull. Ser. F Biotechnol..

[12] Montegiove N., Calzoni E., Cesaretti A., Alabed H., Pellegrino R.M., Emiliani C., Pellegrino A., Leonardi L. (2020). Biogenic Amine Analysis in Fresh Meats and Meat Meals Used as Raw Materials for Dry Pet Food Production. Sci. Bull. Ser. F Biotechnol..

[13] Montegiove N., Calzoni E., Cesaretti A., Pellegrino R.M., Emiliani C., Pellegrino A., Leonardi L. (2021). Soluble Protein Content Assessment in Dry Pet Food Raw Materials: Comparison between Fresh Meat and Meat Meal Formulations. Sci. Bull. Ser. F Biotechnol..

[14] Hamper B.A. (2014). Raw Meat-Based Diets: Current Evidence Regarding Benefits and Risks. A Healthy Begin..

[15] Laflamme D., Izquierdo O., Eirmann L., Binder S. (2014). Myths and Misperceptions about Ingredients Used in Commercial Pet Foods. Vet. Clin. Small Anim. Pract..

[16] Murray S.M., Patil A.R., Fahey G.C., Merchen N.R., Hughes D.M. (1997). Raw and Rendered Animal By-Products as Ingredients in Dog Diets. J. Anim. Sci..

[17] Kies C. (1981). Bioavailability: A Factor in Protein Quality. J. Agric. Food Chem..

[18] Yamka R.M., Jamikorn U., True A.D., Harmon D.L. (2003). Evaluation of Low-Ash Poultry Meal as a Protein Source in Canine Foods. J. Anim. Sci..

[19] Chanadang S., Koppel K., Aldrich G. (2016). The Impact of Rendered Protein Meal Oxidation Level on Shelf-Life, Sensory Characteristics, and Acceptability in Extruded Pet Food. Animals.

[20] Lankhorst C., Tran Q.D., Havenaar R., Hendriks W.H., van der Poel A.F.B. (2007). The Effect of Extrusion on the Nutritional Value of Canine Diets as Assessed by in Vitro Indicators. Anim. Feed Sci. Technol..

[21] Rokey G.J., Plattner B., De Souza E.M. (2010). Feed Extrusion Process Description. Rev. Bras. De Zootec..

[22] Tran Q.D., Hendriks W.H., van der Poel A.F. (2008). Effects of Extrusion Processing on Nutrients in Dry Pet Food. J. Sci. Food Agric..

[23] Williams P.A., Hodgkinson S., Rutherfurd S., Hendriks W. (2006). Lysine Content in Canine Diets Can Be Severely Heat Damaged. J. Nutr..

[24] Johnson M.L., Parsons C.M., Fahey G.C., Merchen N.R., Aldrich C.G. (1998). Effects of Species Raw Material Source, Ash Content, and Processing Temperature on Amino Acid Digestibility of Animal by-Product Meals by Cecectomized Roosters and Ileally Cannulated Dogs. J. Anim. Sci..

[25] Shirley R.B., Parsons C.M. (2001). Effect of Ash Content on Protein Quality of Meat and Bone Meal. Poult. Sci..

[26] Wang X., Parsons C. (1998). Effect of Raw Material Source, Processing Systems, and Processing Temperatures on Amino Acid Digestibility of Meat and Bone Meals. Poult. Sci..

[27] Haines J., Patel M., Knight A.I., Corley D., Gibson G., Schaaf J., Moulin J., Zuber S. (2015). Thermal Inactivation of Feline Calicivirus in Pet Food Processing. Food Environ. Virol..

[28] Okelo P.O., Joseph S.W., Wagner D.D., Wheaton F.W., Douglass L.W., Carr L.E. (2008). Improvements in Reduction of Feed Contamination: An Alternative Monitor of Bacterial Killing During Feed Extrusion1. J. Appl. Poult. Res..

[29] Leiva A., Molina A., Redondo-Solano M., Artavia G., Rojas-Bogantes L., Granados-Chinchilla F. (2019). Pet Food Quality Assurance and Safety and Quality Assurance Survey within the Costa Rican Pet Food Industry. Animals.

[30] Singh S., Gamlath S., Wakeling L. (2007). Nutritional Aspects of Food Extrusion: A Review. Int. J. Food Sci. Technol..

[31] Altan A., McCarthy K.L., Maskan M. (2009). Effect of Extrusion Cooking on Functional Properties and in Vitro Starch Digestibility of Barley-Based Extrudates from Fruit and Vegetable By-Products. J. Food Sci..

[32] Inal F., Alatas M.S., Kahraman O., Inal S., Uludag M., Gurbuz E., Polat E.S. (2018). Using of Pelleted and Extruded Foods in Dog Feeding. Kafkas Univ. Vet. Fak. Derg..

[33] Koppel K., Monti M., Gibson M., Alavi S., Donfrancesco B.D., Carciofi A.C. (2015). The Effects of Fiber Inclusion on Pet Food Sensory Characteristics and Palatability. Animals.

[34] Lin S., Hsieh F., Huff H.E. (1998). Effects of Lipids and Processing Conditions on Lipid Oxidation of Extruded Dry Pet Food during Storage1Contribution from the Missouri Agricultural Experiment Station, Journal Series No. 12590.1. Anim. Feed Sci. Technol..

[35] Hullar I., Fekete S., Szöcs Z. (1998). Effect of Extrusion on the Quality of Soybean-Based Catfood. J. Anim. Physiol. Anim. Nutr..

[36] Alonso R., Aguirre A., Marzo F. (2000). Effects of Extrusion and Traditional Processing Methods on Antinutrients and in Vitro Digestibility of Protein and Starch in Faba and Kidney Beans. Food Chem..

[37] Hendriks W.H., Sritharan K. (2002). Apparent Ileal and Fecal Digestibility of Dietary Protein Is Different in Dogs. J. Nutr..

[38] Rikken G.L.J.A., Raupach E. (2000). Enantioselective Magnetochiral Photochemistry. Nature.

[39] Obrosova I.G., Fathallah L., Stevens M.J. (2001). Taurine Counteracts Oxidative Stress and Nerve Growth Factor Deficit in Early Experimental Diabetic Neuropathy. Exp. Neurol..

[40] Zhang M., Izumi I., Kagamimori S., Sokejima S., Yamagami T., Liu Z., Qi B. (2004). Role of Taurine Supplementation to Prevent Exercise-Induced Oxidative Stress in Healthy Young Men. Amino Acids.

[41] Pion P.D., Kittleson M.D., Rogers Q.R., Morris J.G. (1987). Myocardial Failure in Cats Associated with Low Plasma Taurine: A Reversible Cardiomyopathy. Science.

[42] Morris J.G., Rogers Q.R., Pacioretty L.M. (1990). Taurine: An Essential Nutrient for Cats. J. Small Anim. Pract..

[43] Hayes K.C., Trautwein E.A. (1989). Taurine Deficiency Syndrome in Cats. Vet. Clin. N. Am. Small Anim. Pract..

[44] Knopf K., Sturman J.A., Armstrong M., Hayes K.C. (1978). Taurine: An Essential Nutrient for the Cat. J. Nutr..

[45] Bradford M.M. (1976). A Rapid and Sensitive Method for the Quantitation of Microgram Quantities of Protein Utilizing the Principle of Protein-Dye Binding. Anal. Biochem..

[46] Latimer G.W. (2016). Official Methods of Analysis of AOAC International.

[47] Nielsen S., Food Analysis (2017). Food Science Text Series.

[48] Bradstreet R.B. (1954). Kjeldahl Method for Organic Nitrogen. Anal. Chem..

[49] Jiang B., Tsao R., Li Y., Miao M., Van Alfen N.K. (2014). Food Safety: Food Analysis Technologies/Techniques. Encyclopedia of Agriculture and Food Systems.

[50] Otter D.E. (2012). Standardised Methods for Amino Acid Analysis of Food. Br. J. Nutr..

[51] Adler S.A., Slizyte R., Honkapää K., Løes A.-K. (2018). In Vitro Pepsin Digestibility and Amino Acid Composition in Soluble and Residual Fractions of Hydrolyzed Chicken Feathers. Poult. Sci..

[52] Gehrke C.W., Wall L.L., Absheer J.S., Kaiser F.E., Zumwalt R.W. (1985). Sample Preparation for Chromatography of Amino Acids: Acid Hydrolysis of Proteins. J. Assoc. Off. Anal. Chem..

[53] Pellegrino R.M., Di Veroli A., Valeri A., Goracci L., Cruciani G. (2014). LC/MS Lipid Profiling from Human Serum: A New Method for Global Lipid Extraction. Anal. Bioanal. Chem..

[54] Sud M., Fahy E., Cotter D., Brown A., Dennis E.A., Glass C.K., Merrill A.H., Murphy R.C., Raetz C.R.H., Russell D.W. (2007). LMSD: LIPID MAPS Structure Database. Nucleic Acids Res..

[55] Biagi G., Piva A. (2007). In Vitro Effects of Some Organic Acids on Swine Cecal Microflora. Ital. J. Anim. Sci..

[56] Baker D.H., Czarnecki-Maulden G.L. (1991). Comparative Nutrition of Cats and Dogs. Annu. Rev. Nutr..

[57] Legrand-Defretin V. (1994). Differences between Cats and Dogs: A Nutritional View. Proc. Nutr. Soc..

[58] Massey K.A., Blakeslee C.H., Pitkow H.S. (1998). A Review of Physiological and Metabolic Effects of Essential Amino Acids. Amino Acids.

[59] Morris J.G., Rogers Q.R. (1994). Assessment of the Nutritional Adequacy of Pet Foods through the Life Cycle. J. Nutr..

[60] Gross K.L., Becvarova I., Debraekeleer J. (2010). Feeding Nursing and Orphaned Kittens from Birth to Weaning. Small Animal Clinical Nutrition.

[61] Nie C., He T., Zhang W., Zhang G., Ma X. (2018). Branched Chain Amino Acids: Beyond Nutrition Metabolism. Int. J. Mol. Sci..

[62] Shimomura Y., Murakami T., Nakai N., Nagasaki M., Harris R.A. (2004). Exercise Promotes BCAA Catabolism: Effects of BCAA Supplementation on Skeletal Muscle during Exercise. J. Nutr..

[63] Calders P., Matthys D., Derave W., Pannier J.L. (1999). Effect of Branched-Chain Amino Acids (BCAA), Glucose, and Glucose plus BCAA on Endurance Performance in Rats. Med. Sci. Sports Exerc..

[64] Rieu I., Sornet C., Bayle G., Prugnaud J., Pouyet C., Balage M., Papet I., Grizard J., Dardevet D. (2003). Leucine-Supplemented Meal Feeding for Ten Days Beneficially Affects Postprandial Muscle Protein Synthesis in Old Rats. J. Nutr..

[65] Proud C.G. (2002). Regulation of Mammalian Translation Factors by Nutrients. Eur. J. Biochem..

[66] Brown R.G. (1989). Protein in Dog Food. Can. Vet. J..

[67] Case L., Daristotle L., Hayek M., Raasch M. (2011). Canine and Feline Nutrition.

[68] Milner J.A. (1981). Lysine Requirements of the Immature Dog. J. Nutr..

[69] Rutherfurd-Markwick K.J., McGrath M.C., Weidgraaf K., Hendriks W.H. (2006). γ-Glutamylfelinylglycine Metabolite Excretion in the Urine of the Domestic Cat (Felis Catus). J. Nutr..

[70] Burns R.A., Milner J.A. (1981). Sulfur Amino Acid Requirements of Immature Beagle Dogs. J. Nutr..

[71] Teeter R.G., Baker D.H., Corbin J.E. (1978). Methionine and Cystine Requirements of the Cat. J. Nutr..

[72] Hendriks W.H., Rutherfurd S.M., Rutherfurd K.J. (2001). Importance of Sulfate, Cysteine and Methionine as Precursors to Felinine Synthesis by Domestic Cats (Felis Catus). Comp. Biochem. Physiol. Part C Toxicol. Pharmacol..

[73] (2013). EFSA Panel on Additives and Products or Substances used in Animal Feed (FEEDAP) Scientific Opinion on the Safety and Efficacy of L-Tyrosine for All Animal Species. EFSA J..

[74] Morris J.G., Yu S., Rogers Q.R. (2002). Red Hair in Black Cats Is Reversed by Addition of Tyrosine to the Diet. J. Nutr..

[75] Anderson P.J.B., Rogers Q.R., Morris J.G. (2002). Cats Require More Dietary Phenylalanine or Tyrosine for Melanin Deposition in Hair than for Maximal Growth. J. Nutr..

[76] Burns R.A., Milner J.A. (1982). Threonine, Tryptophan and Histidine Requirements of Immature Beagle Dogs. J. Nutr..

[77] Titchenal C.A., Rogers Q.R., Indrieri R.J., Morris J.G. (1980). Threonine Imbalance, Deficiency and Neurologic Dysfunction in the Kitten. J. Nutr..

[78] Iwazaki E., Nade T., Kimura N. (2019). Effects of Overfeeding on the Fatty Acid Profile and Stearoyl-CoA Desaturase-1 Indices in the Liver and Subcutaneous Adipose Tissue in Cats. J. Vet. Med. Sci..

[79] Ahlstrøm Ø., Krogdahl Å., Vhile S.G., Skrede A. (2004). Fatty Acid Composition in Commercial Dog Foods. J. Nutr..

[80] Butterwick R.F., Salt C., Watson T.D.G. (2012). Effects of Increases in Dietary Fat Intake on Plasma Lipid and Lipoprotein Cholesterol Concentrations and Associated Enzyme Activities in Cats. Am. J. Vet. Res..

[81] Grundy S.M. (1994). Influence of Stearic Acid on Cholesterol Metabolism Relative to Other Long-Chain Fatty Acids. Am. J. Clin. Nutr..

[82] Sassa T., Kihara A. (2014). Metabolism of Very Long-Chain Fatty Acids: Genes and Pathophysiology. Biomol. Ther..

[83] Li Z., Zhang Y., Su D., Lv X., Wang M., Ding D., Ma J., Xia M., Wang D., Yang Y. (2014). The Opposite Associations of Long-Chain versus Very Long-Chain Monounsaturated Fatty Acids with Mortality among Patients with Coronary Artery Disease. Heart.

[84] Yaqoob P. (2002). Monounsaturated Fatty Acids and Immune Function. Eur. J. Clin. Nutr..

[85] Palmquist D.L. (2009). Omega-3 Fatty Acids in Metabolism, Health, and Nutrition and for Modified Animal Product Foods. Prof. Anim. Sci..

